# Dr. Mütter’s Donation

**Published:** 1859-03

**Authors:** 


DR. MUTTER'S DONATION TO THE COLLEGE OF PHYSICIANS OF PHILADELPHIA.—Early in January last, Dr. T. D. Mütter perfected his agreement with the College of Physicians, by which he gives to that institution his museum, together with his plates and diagrams, to serve as a basis for the "Mütter Museum." Besides this, he endows the college with thirty thousand dollars, to defray the expenses of the museum and to establish lectureships. The College is bound, on its part, to have a fire-proof building completed within five years, for the accommodation of the collection.

